# A Zebrafish Larval Model to Assess Virulence of Porcine *Streptococcus suis* Strains

**DOI:** 10.1371/journal.pone.0151623

**Published:** 2016-03-21

**Authors:** Edoardo Zaccaria, Rui Cao, Jerry M. Wells, Peter van Baarlen

**Affiliations:** Host-Microbe Interactomics, Department of Animal Sciences, Wageningen University, Wageningen, the Netherlands; Instituto Butantan, BRAZIL

## Abstract

*Streptococcus suis* is an encapsulated Gram-positive bacterium, and the leading cause of sepsis and meningitis in young pigs resulting in considerable economic losses in the porcine industry. It is also considered an emerging zoonotic agent. In the environment, both avirulent and virulent strains occur in pigs, and virulent strains appear to cause disease in both humans and pigs. There is a need for a convenient, reliable and standardized animal model to assess *S*. *suis* virulence. A zebrafish (*Danio rerio*) larvae infection model has several advantages, including transparency of larvae, low cost, ease of use and exemption from ethical legislation up to 6 days post fertilization, but has not been previously established as a model for *S*. *suis*. Microinjection of different porcine strains of *S*. *suis* in zebrafish larvae resulted in highly reproducible dose- and strain-dependent larval death, strongly correlating with presence of the *S*. *suis* capsule and to the original virulence of the strain in pigs. Additionally we compared the virulence of the two-component system mutant of *ciaRH*, which is attenuated for virulence in both mice and pigs *in vivo*. Infection of larvae with the Δ*ciaRH* strain resulted in significantly higher survival rate compared to infection with the S10 wild-type strain. Our data demonstrate that zebrafish larvae are a rapid and reliable model to assess the virulence of clinical porcine *S*. *suis* isolates.

## Introduction

*Streptococcus suis* is a zoonotic Gram-positive bacterial pathogen and the leading cause of sepsis and meningitis in young pigs. Infections with *S*. *suis* occur worldwide throughout pig production industry, resulting in considerable economic losses [1, 2]. In recent years *S*. *suis* isolates causing more rapid and severe infections in both humans and pigs have been identified leading to concerns about the emergence of more virulent strains [3, 4]. At least 35 different serotypes of the capsular polysaccharide have been identified of which serotype 2 is the most frequently isolated from infected pigs and humans [4, 5]. In addition, serotypes 1, 9, and 14 have often been associated with porcine disease [6, 7]. Its natural habitat is the upper respiratory tract of pigs, the tonsil and nasal cavity in particular [8] and the intestine [9]. Healthy pigs that carry *S*. *suis* are a source for its transmission to the herd [8]. At present, *S*. *suis* is the most common cause of adult bacterial meningitis in Vietnam [10] and the second most common in Thailand [11]. Moreover, two human outbreaks with high mortality rates have been reported in China [5, 12, 13] underlining its importance as a zoonotic agent in Asia. In Europe, the largest number of zoonotic infections due to *S*. *suis*, have been recorded in the Netherlands (Fig 1 in [5]). In pigs and humans, meningitis, endocarditis and streptococcal toxic shock-like syndrome are the most common symptoms caused by virulent *S*. *suis* strains [14–16]. Currently it is impossible to assess the virulence of an *S*. *suis* strain using molecular markers [17] highlighting the need for better genomic markers and animal models to establish the genetic determinants of virulence in different isolates.

Notwithstanding its prominence as a zoonotic agent, little is known about *S*. *suis* pathogenicity, virulence and mechanism of infection. Genetic analysis of virulence and pathogenicity is challenging because *S*. *suis* produces multifactorial virulence factors [17] and because natural populations are characterized by high rates of recombination [18, 19], creating many different genotypes of which only few have been well characterized for virulence [1]. One generally accepted virulence factor of *S*. *suis* is the capsular polysaccharide, and its critical role in virulence and pathogenicity has been demonstrated in multiple independent studies [20–22]. However, unencapsulated strains have been isolated from pigs with invasive disease [23].

A simple and reliable animal model would be a valuable tool to assess virulence of natural isolates of *S*. *suis* and establish the role of genetic determinants in virulence. Pigs and mice have been successfully employed for virulence studies [24, 25], but have economical, logistic and ethical disadvantages over non-mammalian models, including nematodes and zebrafish (*Danio rerio*). Adult zebrafish have been used as an infection model to study *S*. *suis* with some success [26, 27], however these studies used only one or two strains and did not assess the usefulness of the model to predict strain virulence in pigs or humans. In addition, adult zebrafish are not exempt from ethical legislation. Pre-feeding zebrafish larvae (up to 6 days post fertilization) are exempt from ethical legislation and cheap to rear in large numbers, allowing high-throughput screens to be performed that would be much less feasible in experimental mammalian models of infection. We were thus interested to evaluate if pre-feeding-stage zebrafish larvae could be used as an animal model to assess virulence of natural porcine *S*. *suis* isolates.

Zebrafish is a teleost fish of the Cyprinidae family. The zebrafish embryonal and larval innate immune system develops rapidly and functional phagocytes, complement factors, and antimicrobial enzymes are present in the embryo before or soon after hatching [28–31]. Also granuloma-like structures, the result of macrophage aggregation, do occur during infection in zebrafish larvae [32, 33] showing that the larval immune system is competent to provide resistance against bacterial infection. Zebrafish larvae have been used extensively as an animal infection model for a wide range of Gram-negative and Gram-positive bacterial pathogens [28, 34]. *Salmonella*, *Mycobacterium marinum* and *Listeria monocytogenes* infection studies, as well as *Streptococcus* species, have been successfully accomplished using this model organism [34–38].

To date, *S*. *suis* infection of zebrafish larvae has not been explored. Theoretically, using larvae has several advantages over using adult fish. Short-term (1–3 days) maintenance and handling of larvae in the laboratory environment is low-cost and easy, and their transparency allows various optical microscopy and imaging technologies at high resolution. As mentioned above, larvae 24–48 hours post-fertilization (hpf), already carry a mature innate immune system [39].

Here we examined *S*. *suis* infection in zebrafish larvae at 72 hpf. We found that zebrafish larvae are susceptible to infection by virulent porcine *S*. *suis* strains belonging to different serotypes and determined the median lethal dose (LD_50_). Importantly, bacterial isolates reported as weakly virulent, and unencapsulated mutants were attenuated in the larval model. These findings propose a novel system to assess *S*. *suis* virulence using a convenient, cost-effective and reproducible zebrafish larval infection model.

## Materials and Methods

### Bacterial strains, plasmids and culture conditions

The *S*. *suis* strains described in this manuscript are listed in Table 1. *S*. *suis* strains were grown in Todd-Hewitt Broth (THB) (Difco, USA) at 37°C, in the presence of 5% CO_2_. When required, spectinomycin (100 μg/ml) was added to the media to select for genetically modified strains. Solid agar plates were prepared by adding 12 g/L agar to the medium. Overnight cultures were diluted 1:100 in fresh THB and grown until exponential phase (optical density at 600nm ca 0.4) and stored at -80°C in 15% of glycerol. After thawing bacterial colony forming units (CFU) were determined by plating on THB agar.

**Table 1:** *S*. *suis* strains used in this study and their virulence. V, virulent; WV, weakly virulent; AV, avirulent.

*S*. *suis* strain	Serotype	Virulence in pigs	Reference
S10	2	V	[20]
S10-J28	2	WV	[20]
p1/7	2	V	[40]
6555	1	WV	[41]
6388	1	V	[42]
5218	9	AV	[41]
8067	9	AV	[43]
S735	2	WV	[44]

### Zebrafish stock and larvae

Zebrafish embryos were obtained from breeders of the Zod2F8 line and were bred at the animal facility of Wageningen University. In the animal facility, a female zebrafish can lay up to 200 eggs a week. Ca. 60 eggs/dish were kept in oxygenated sterile egg water (60 μg/mL Sea salts) at 28°C. Debris and unfertilized eggs were removed twice a day until hatching. Larvae were sacrificed by an overdose of the anesthetic 3-aminobenzoic acid (Tricaine, Sigma-Aldrich, USA) buffered with 1.5% NaHCO_3_ at 50 hours post infection (hpi). All zebrafish were raised, maintained, and handled in compliance with the local animal welfare regulations, and according to standard protocols (zfin.org) under the guidelines of Wageningen University and Research Centre Institutional Animal Care and Use Committee.

### Bacterial staining

Overnight cultures of *S*. *suis* S10 were diluted 1:100 in fresh THB, grown until exponential phase and collected by centrifugation (5000 g, 10 min). The pellet was resuspended in 5 μm CFSE in PBS buffer and incubated in the dark for 30 minutes. Bacteria were washed in PBS three times, diluted and use for microinjection. For each condition (microinjection into the Duct of Couvier, yolk sac and controls group), at 1 hpi, the viability of 10 anesthetized larvae was visually checked using a fluorescence stereo-microscope (M205 FA, Leica).

### Zebrafish microinjection

Zebrafish larvae were anesthetized with 200 μg/mL 3-aminobenzoic acid approximately 10 min prior to injection. The larvae were kept in a Petri dish filled with egg water and with a layer of 1% agarose on the bottom. The larvae were checked for blood circulation consistency under a stereo-microscope and vital larvae were injected with 1 nl of bacterial suspension into the yolk sac or into the Duct of Cuvier (DoC). Prior to injection, bacteria were obtained by centrifugation (5000 rpm, 5 minutes) and resuspended in 2% polyvinylpyrrolidone (PVP) to avoid clogging of the ultrafine needle and to maintain a homogeneous bacterial distribution. To determine the bacterial count, 1 nl of bacterial suspension was added to 100 μl of sterile PBS, diluted and plated in THB agar plates with the required antibiotics. To obtain an optimal number of bacteria per inoculum, total numbers of 100, 500, 1000, 2500 and 5000 CFU were initially tested. At 48 hpf, 25 larvae per condition, per strain were collected using filtered water and sterile Petri dishes under aseptic conditions in a flow cabinet. Experiments were performed in triplicate.

### Statistical analysis

GraphPad Prism version 6.0c was used for statistical analysis. A paired T-test was used to determine significant differences between *S*. *suis* growth curves at 28°C and at 37°C. Significant differences between mortality rates were determined by the Kruskal-Wallis test, and the Mantel-Cox test was used to determine significant differences between survival curves. Statistical significance was accepted at *p* < 0.05.

## Results

### *S*. *suis* grows efficiently at 28°C

**Fig 1:**
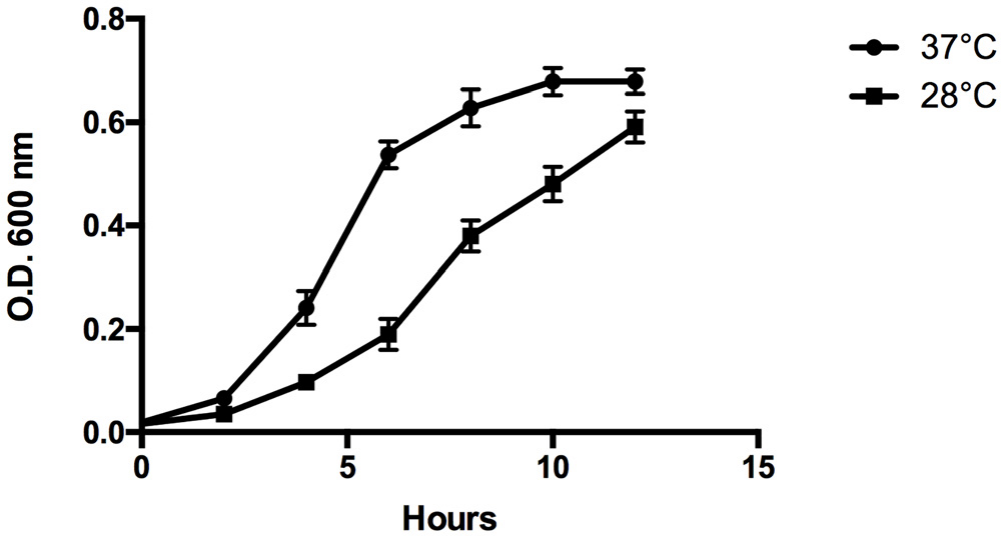
*S*. *suis g*rowth curves at 28°C and at 37°C. 1 ml of culture was sampled and absorbance (or optical density) at 600 nm (OD_600_) was measured every 2 hours; the difference between the two growth curves was weakly significant (*p* = 0.0172).

To determine the ability of *S*. *suis* to efficiently grow at 28°C, the rearing temperature of zebrafish larvae, an overnight culture was diluted and incubated at 28°C and at 37°C, both at 5% of CO_2_. We sampled 1 ml of both cultures every 2 hours and measured their absorbance at 600 nm. At 28°C *S*. *suis* grows to almost the same final OD_600nm_ as cultures incubated at 37°C but the lag phase was longer and the growth rate slightly slower than at 37°C (Fig 1); this difference was weakly significant (*p* = 0.0172).

### Localization of fluorescent *S*. *suis* in injected zebrafish larvae

**Fig 2:**
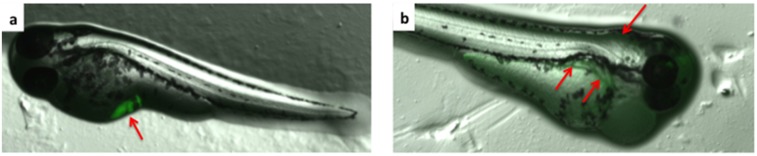
Fluorescent *S*. *suis* in injected zebrafish larvae. Zebrafish larvae were injected with 1000 colony forming units in the yolk sac and in the Duct of Couvier (DoC) and observed under a Leica M205 FA fluorescent stereo-microscope. a) Localization of fluorescent *S*. *suis* S10 injected in the yolk sac, 1 hpi, b) Localization of fluorescent *S*. *suis* S10 injected in the DoC, 1 hpi.

In order to ensure the successful injection of *S*. *suis* into the zebrafish larvae, visual tracking of injected bacteria was performed. Carboxyfluorescein succinimidyl ester (CFSE), a dye that readily crosses intact cell membranes, was used to stain bacterial cells. Once inside the cells, intracellular esterases cleave acetate groups to yield the fluorescent carboxyfluorescein molecule. The succinimidyl ester group reacts with primary amines, crosslinking the dye to intracellular proteins such that the dye is restrained within the bacteria. CFSE-stained *S*. *suis* S10 was injected in the yolk sac and in the Duct of Cuvier (DoC) and the zebrafish larvae were observed via a fluorescence stereo microscope (M205 FA, Leica) at 1 hpi. Bacteria were visible in the yolk sac and in the bloodstream (green fluorescent signals in Fig 2a and 2b) confirming successful injection and showing that bacteria injected in the DoC injection become efficiently dispersed via the bloodstream to blood vessels.

### Microinjection of zebrafish larvae with *S*. *suis* S10 is fatal and dose-dependent

**Fig 3:**
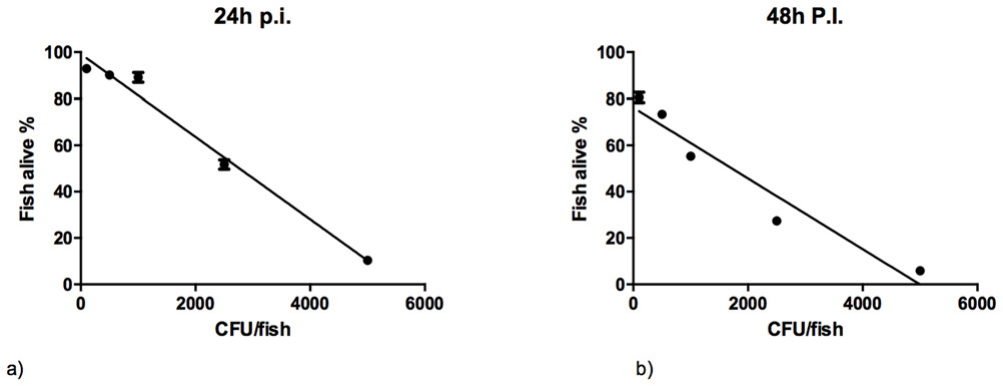
LD_50_ determination. Zebrafish larvae were injected with 100, 500, 1000, 2500 and 5000 colony forming units. a. Survival rate of infected larvae at 24 hpi. b. Survival rate of infected larvae at 48 hpi. At both time points, results were obtained in 3 independent experiments, and 25 larvae were injected per group.

To determine the susceptibility of zebrafish larvae to *S*. *suis* infection and establish lethal dosages, the virulent serotype 2 isolate S10 was microinjected into the yolk sac of the larvae at 72 hpf. Zebrafish larvae were injected with 100, 500, 1000, 2500 and 5000 colony forming units (CFU) resuspended in polyvinylpyrrolidone (PVP). The control group was injected with PVP only. Each group contained 25 larvae per condition, and each experiment was performed in triplicate. The LD_50_ for larval death after injection of strain S10 was determined at 24 and 48 hpi and shown to be dose-dependent. At 24 hpi the LD_50_ was 2.7*10^3^ CFU and at 48 hpi 1.6*10^3^ CFU per larva (Fig 3a and 3b).

### Zebrafish larval mortality depends on the virulence of the injected strain

**Fig 4:**
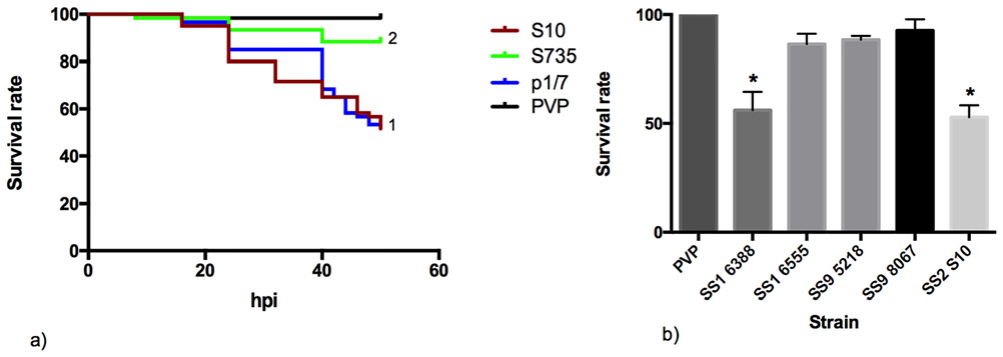
Zebrafish larval mortality correlates with virulence of the injected *S*. *suis* strain in pigs. a. Survival curve of yolk-injected larvae with three different *S*. *suis* serotype 2 strains. Larval viability was checked every 8h for the first 40h, then every 2h. (1) *p* < 0.001 compared with S735 and PVP control curves. (2) *p* < 0.01 compared with S10, P1/7 and PVP control group. b. Survival rate of yolk-injected larvae with different *S*. *suis* strains; larval survival was determined at 48 hpi. * *p* < 0.001 compared with SS1 6555, SS9 5218, SS9 8067 and PVP control group. Results were obtained in 3 independent experiments, and 25 larvae were injected per group.

To assess the accuracy of the zebrafish larvae infection model for predicting the virulence of *S*. *suis* strains in pigs, we tested strains with known differential virulence in pigs using the same bacterial inoculum for each strain (1.6*10^3^ ± 100). Yolk injection with strain S735, reported as weakly virulent [42, 44], showed a mortality of 10% at 48 hpi (*p* < 0.01 compared with S10, P1/7 and the PVP control group). In contrast, the virulent strain S10 caused nearly 50% of larval death after 48 hours. The zebrafish larvae infected with the virulent strain p1/7 [40] also resulted in about 50% larval mortality (for both virulent strains *p* < 0.001 when compared with S735 and PVP control group). No significant difference was found between mortality of larvae infected with virulent strains p1/7 or S10 (p > 0.05) (Fig 4a).

Infection with serotype 9 strains 8067 and 5218, reported as avirulent and weakly virulent porcine strains respectively [45], resulted in very low mortality rates of 7.5% for 8067 and 11.6% for 5218. Serotype 1 strain 6388, which has been described as a hypervirulent porcine strain [42], caused nearly 50% of larval death at 48 hpi (Fig 4b) (*p* < 0.001 compared with the PVP control group and with the other *S*. *suis* strains except S10). In comparison, serotype 1 strain 6555, reported as weakly virulent [41] showed no significant difference in larval killing compared to the PVP control group with a mortality rate of 12.2% (Fig 4b).

### Microinjection of *S*. *suis* in the zebrafish yolk sac or the Ducts of Cuvier leads to similar rates of mortality

**Fig 5:**
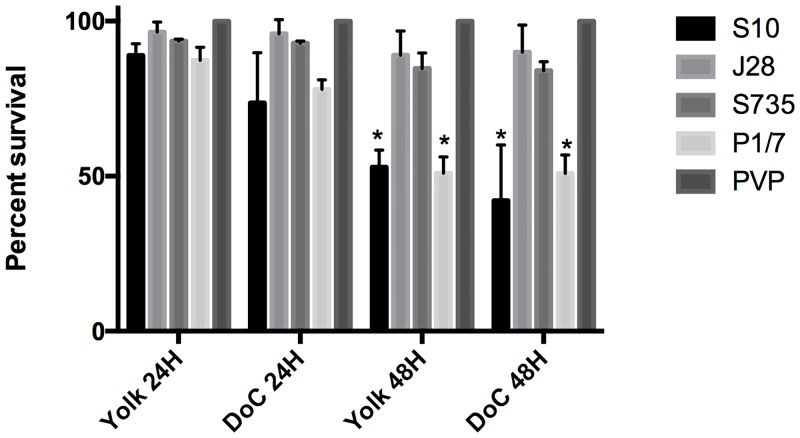
Microinjection in the yolk sac or the ducts of Cuvier leads to similar mortality rates. Survival rate of injected larvae with four different *S*. *suis* strains in two different infection sites, the yolk sac and the Ducts of Cuvier, no significant difference between the two infection sites was found (*p* = 0.0536). The surviving larvae were counted at 24 and 48 hpi. * P-value < 0.01 compared with J28, S735 and PVP control group. Results were obtained in 3 independent experiments, and 25 larvae were injected per group.

In pigs, the most serious complications upon *S*. *suis* infections derive from bloodstream infections. In zebrafish larvae, injections into the bloodstream are technically more challenging than injection into the yolk sac. To evaluate if injections into the bloodstream or yolk sac resulted in differential killing of zebrafish larvae, the same dose (1.6*10^3^ ± 100) of four *S*. *suis* serotype 2 strains were injected in the yolk sac and in the Ducts of Cuvier (DoC), the common cardinal vein that directs the blood that returns from the tail regions back to the heart. Percentage of survival was determined at 24 hpi and 48 hpi. Virulent strains caused 50% of larval death at 48 hpi, with a minor increase in mortality when the bacteria were inoculated directly into the bloodstream; no significant difference between mortality upon injection in the two organs was found (*p* = 0.0536). As in the previous infection assays, infection with weak or avirulent strains resulted in very low mortality (Fig 5), with no significant difference with the control group. These data show that injection into the readily accessible yolk sac leads to comparable larval death rates as compared to injection into the DoC, which is technically more difficult to access.

### Capsular polysaccharide is an important virulence factor for *S*. *suis* infection of zebrafish larvae

**Fig 6:**
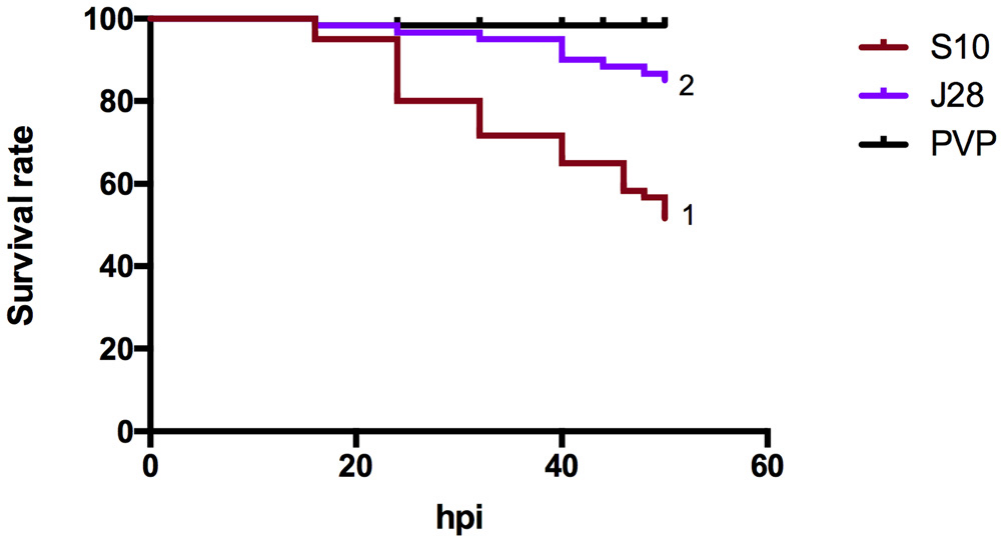
Relevance of the capsular polysaccharide during infection of zebrafish larvae. Survival curve of injected larvae with *S*. *suis* S10, its unencapsulated mutant strain J28 and PVP control. Larval viability was checked every 8h for the first 40h, then every 2h. (1) *p* < 0.001 compared with J28 and PVP control curves. (2) *p* < 0.01 compared with S10 and PVP control curves. Results were obtained in 3 independent experiments, and 25 larvae were injected per group.

In a previous study, an unencapsulated mutant was reported to be less infectious in pigs [46] and to be phagocytized by human and porcine dendritic cells at higher rates than the corresponding wild-type strain S10 [21, 22]. To investigate if the same difference in virulence could be observed during infection of zebrafish larvae, *S*. *suis* strain S10 and the unencapsulated mutant J28 were injected using the same bacterial load (1.6*10^3^ ± 100 CFU/larvae) into the yolk sac and the infection was monitored for 48h. Infection of zebrafish larvae by the unencapsulated J28 mutant resulted in a significant decrease in mortality compared with the S10 wild-type strain, with survival rates of 85.1% and 50.3% respectively (*p* < 0.001), thus showing the same differential infection capacity as previously found in controlled pig infections (Fig 6).

### Deletion of the two-component system *ciaRH* reduced *S*. *suis* virulence in zebrafish larvae

**Fig 7:**
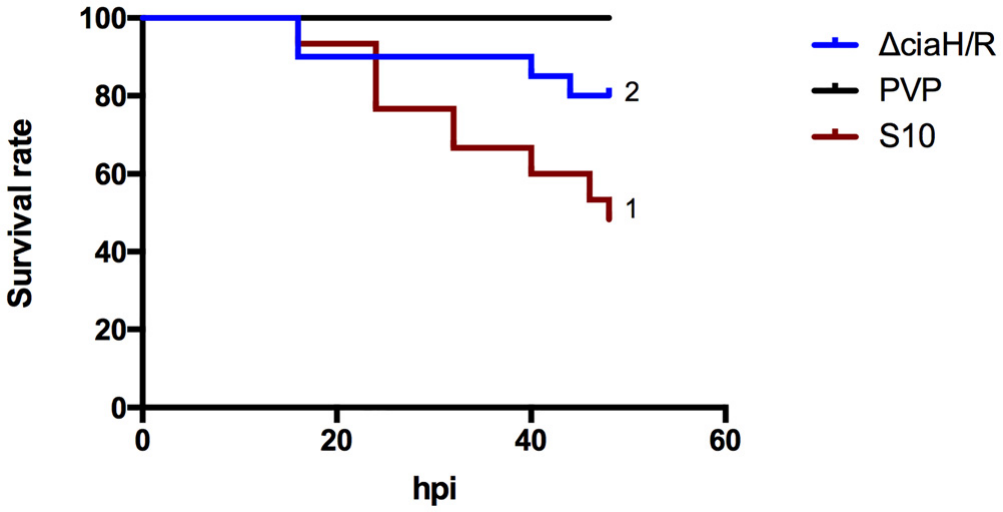
Increased survival rate of zebrafish larvae injected with S10 ΔciaRH compared with the wild-type strain. Survival curve of injected larvae with S. suis S10, its mutant strain ΔciaRH and control. Larval viability was checked every 8h for the first 40h, then every 2h. (1) *p* < 0.01 compared with ΔciaRH and PVP control curves. (2) *p* < 0.01 compared with S10 and PVP control curves. Results were obtained in 3 independent experiments, and 25 larvae were injected per group.

In a previous study Li *et al* [47] showed the relevant contribution to the virulence of *S*. *suis* serotype 2 of the two component system *ciaRH*. The deletion of this operon resulted in a lower mutant survival rate in a bactericidal assay compared to the wild-type. Moreover, the mutant was attenuated for virulence in both mice and pigs *in vivo*, as indicated by lower mortality and morbidity. In order to assess whether the same reduced virulence of *S*. *suis* strain S10 Δ*ciaRH* would be observed in zebrafish larvae, 1.6*10^3^ ± 100 CFU/larvae of the mutant and corresponding wild-type strain were separately injected into the yolk sacs of groups of zebrafish larvae and mortality monitored for 48h. Infection of larvae by the Δ*ciaRH* strain resulted in significantly higher survival rate compared to infection with the S10 wild-type strain, 79.8% and 48.9% respectively (*p* < 0.001) (Fig 7), thus reproducing previous results obtained with the same two isolates in mouse and pig infection studies. Taken together these results show that the virulence of *S*. *suis* strains in experimental pig infections is reflected in the zebrafish larvae infection model, inferring that zebrafish larvae can be used to reproducibly assess virulence of porcine *S*. *suis* strains, in a biologically meaningful way.

## Discussion

Given the societal importance of *Streptococcus suis* infections in pigs and humans, and the need for epidemiological markers of strain virulence and the notion that virulent isolates can infect both pigs and humans, there is an urgent need for a standardized and cost-effective measure of relative virulence. *S*. *suis* infection studies using adult zebrafish have been performed successfully in the past to assess virulence of specific strains and mutants [27, 48–51] but this has some disadvantages compared to the zebrafish larval model. Adult zebrafish are more expensive to rear than larvae due to the need for long-term maintenance and aquaria for experimentation. Unlike larvae in the first few days of their life, adult zebrafish are not transparent, precluding the use of high-resolution optical microscopy and imaging technologies. Published studies indicate that number of CFU of *S*. *suis* that need to be injected to cause 50% mortality in adult zebrafish is relatively high and not consistent between laboratories. For example, in one study the median LD_50_ for *S*. *suis* strain HA9801 was 3.8*10^4^ [49] and in a second study it is was reported to be more than 10 fold higher [50].

As zebrafish larvae models have been used to study the pathogenesis and virulence mechanisms of several different pathogens [34–37] we evaluated their potential to assess virulence of porcine *S*. *suis* isolates. Complement components are transferred from the mother to the eggs and effective innate immune responses against microbial challenges, including functional macrophages, can be seen at 24 hours post fertilization (hpf) [30, 52, 53]. At 48 hpf the first granulocytes are visible [54] and at 72 hpf the innate immune system is fully functional [39]. Therefore, we chose the developmental stage at 72 hpf to conduct our experiments. To ensure reproducibility and standardization, we found that it is crucial to infect zebrafish larvae at the same developmental stage. Zebrafish embryos develop very rapidly, and larvae that differ 4–8 hours of age can differ in development [39].

In nature, zebrafish pathogens may enter and infect their hosts in numerous ways, via the gastrointestinal tract, the gills or open wounds. Accordingly, in preliminary experiments, we wanted to evaluate the ability of *S*. *suis* to successfully infect zebrafish larvae in immersion assays where bacteria were added to the water containing the larvae. However, despite the fact that immersion assays showed the natural ability of *S*. *suis* to infect and cause death of zebrafish larvae, we found that this infection method is less reproducible and less consistent than microinjection, as had also been reported by others. Thus microinjection was selected as the preferred mode of infection in subsequent experiments.

To evaluate if the zebrafish larval infection model could quantitatively assess virulence of *S*. *suis* strains, we selected *S*. *suis* serotype 2 strain S10, a porcine highly virulent strain [41], at doses of 100, 500, 1000, 2500 and 5000 CFU by injection into the yolk sac. We noted that the larval mortality rates were dose dependent, ranging from 6.9% of larval death at the lower dose at 24 hpi to 95% at the higher dose at 48 hpi. The LD_50_ values for larvae at 24 and 48 hpi were calculated as 2.7*10^3^ and 1.6*10^3^ CFU per larvae, respectively. These values were comparable to those obtained for other pathogens in the zebrafish larvae model [34, 55, 56]. Furthermore, the results were highly reproducible: we injected more than 500 larvae in independent experiments using the same bacterial load (1.6*10^3^ ± 100) and always obtained the same virulence ranking of strains.

To further characterise the zebrafish larval model to assess virulence of *S*. *suis* strains, we compared larval death rates after infection via two injection sites: the yolk sac and the Duct of Cuvier (DoC), a major blood vessel connecting the heart to the trunk blood circulation system that can be used as an infection site [57]. The yolk sac is a convenient and easy injection site; it has previously been used to achieve systemic infection of slow growing bacteria [58]. However injection with fast growing bacteria can lead to rapid bacterial proliferation and rapid death of the larvae. In pigs, dissemination of *S*. *suis* via the blood to peripheral organs and the brain is a relevant virulence trait [17]. We were therefore interested to compare death rates of zebrafish after injection of two different *S*. *suis* strains into the zebrafish bloodstream or yolk sac. No significant differences (*p* > 0.05) were obtained for the two infection routes using zebrafish larvae from the same egg batch. Thus for convenience, most virulence assays were performed by microinjection into the yolk sac.

To further evaluate the usefulness of a zebrafish larvae infection model for assessing the *in vivo* virulence of porcine *S*. *suis* strains, we tested the virulence of seven clinically relevant porcine *S*. *suis* strains, comprising three serotypes that had been previously studied in porcine infections. Initially, we infected zebrafish larvae with two serotype 2 strains reported as virulent (p1/7) and weakly virulent (S735) [44]. Both strains induced increased mortality rates of zebrafish larvae compared to PVP-injected larvae (*p* < 0.01). At the same dose, the virulent strain p1/7 caused larval death at a similar rate to S10, while S735 infection resulted in a very low larval mortality. Next, we sought to determine death rates induced by other *S*. *suis* serotypes in zebrafish larvae. Two strains of serotype 1, and two strains of serotype 9 with different pathogenicity in pigs were selected for injection. As predicted the virulent pig strain 6388 caused a high mortality rate in zebrafish, whereas mortality rates induced by the two avirulent (strains 5218 and 8067) and a weakly virulent pig strain 6555 were not significantly different to the control group. The virulence of seven natural *S*. *suis* strains and one weakly virulent mutant, S10-J28, in the zebrafish larvae infection model showed consistency with the reported pig virulence studies (Table 2) apart from the isolate 5218. This strain, the reference isolate for serotype 9, is of unknown origin and reported to be avirulent, based on a single piglet infection study [45]. This strain contains a prophage that can be activated *in vitro* [59] and it is possible that this prophage differentially modulates virulence depending on changes in environmental conditions, a common feature of prophages that use bacterial pathogens as host [60].

**Table 2:** *S*. *suis* isolates and their relative virulence in zebrafish larvae and in pig infection studies.

*S*. *suis* strain	Serotype	Origin	Virulence in pigs	Virulence in zebrafish larvae	Reference	Suilysin gene*
**S10**	2	Tonsil	V	V	[41]	+
**S10-J28**	2	Laboratory	WV	WV	[41]	+
**p1/7**	2	Organs	V	V	[40]	+
**6555** ^**R**^	1	Unknown	WV	WV	[45]	ND
**6388**	1	Organs	V	V	[42]	+
**5218** ^**R**^	9	Unknown	AV	WV	[45]	ND
**8067**	9	CNS	AV	AV	[43]	+
**S735**	2	Lungs	WV	WV	[44]	+

*+, present in the genome; ND, not determined; ^**R**^, reference strain for the corresponding serotype.

Next, we tested whether the capsular polysaccharide, a well-characterized *S*. *suis* virulence factor that contributes to disease pathogenesis in porcine infection models, also plays the same important role in infection of zebrafish larvae. We used the unencapsulated mutant J28 that is less virulent [20, 45] and phagocytized more efficiently than the virulent parent strain S10 by human and porcine dendritic cells [21, 22]. In agreement with previous studies, infection of zebrafish larvae with the J28 strain resulted in significantly lower mortality rates compared to infection by the wild-type strain (*p* < 0.01). This result shows that capsule, a confirmed *S*. *suis* virulence factor in pigs, is also required for full virulence in zebrafish larvae, lending further support to the reliability of the zebrafish larval model to assess virulence of porcine *S*. *suis* strains.

We also investigated the impact of the deletion of *ciaRH* on *S*. *suis* serotype 2 virulence. The *ciaHR* operon encodes a two-component system (TCS) consisting of a histidine kinase receptor and a cognate response regulator. TCSs play fundamental roles in regulating bacterial gene expression in response to environmental stimuli. The *ciaHR* TCS contributes to *in vivo S*. *suis* virulence [47]. In a bactericidal assay, using a macrophage cell line, deletion of this TCS resulted in a lower survival rate compared to the wild-type. Moreover, survival rates of mice and pigs infected with the *ciaHR* deletion mutant strain were higher than for the wild-type bacteria. The *ciaHR* mutant was also significantly attenuated in the zebrafish larvae model causing larval mortality of 20% at 48 hpi, compared to 50% mortality with the parent strain. This finding is of interest as it suggests that this TCS is necessary for full virulence in three diverse hosts, and that this TCS may therefore also be necessary for virulence in humans. The *ciaHR* TCS may be an antimicrobial target to treat animal and human infections.

In conclusion we show for the first time that it is possible to use a zebrafish larvae model to assess the relative virulence of porcine *S*. *suis* strains. By exploiting the favorable features of the breeding and maintenance of zebrafish larvae, the number of experiments can be easily scaled-up and results can be obtained within days. Because of its convenience and cost-effectiveness, this model may be used to assay virulence of environmental *S*. *suis* strains that are ubiquitous in pig husbandry and may infect a variety of animals, with most clinical relevance to infection of pigs and humans. Furthermore, a large number of bacterial mutants and strains can be screened for their virulence and *in vivo* pathogenicity, opening up new avenues to investigate the so far undiscovered pathways mediating successful host infection by *S*. *suis*.
